# Mechanism of Ganglioside Receptor Recognition by Botulinum Neurotoxin Serotype E

**DOI:** 10.3390/ijms22158315

**Published:** 2021-08-02

**Authors:** Geoffrey Masuyer, Jonathan R. Davies, Pål Stenmark

**Affiliations:** 1 Department of Biochemistry and Biophysics, Stockholm University, 10691 Stockholm, Sweden; jonathan.davies@dbb.su.se; 2 Centre for Therapeutic Innovation, Department of Pharmacy and Pharmacology, University of Bath, Bath BA2 7AY, UK; 3 Department of Experimental Medical Science, Lund University, 22184 Lund, Sweden

**Keywords:** Clostridium botulinum, botulinum neurotoxin, BoNT/E, gangliosides, glycan binding, receptor binding

## Abstract

The botulinum neurotoxins are potent molecules that are not only responsible for the lethal paralytic disease botulism, but have also been harnessed for therapeutic uses in the treatment of an increasing number of chronic neurological and neuromuscular disorders, in addition to cosmetic applications. The toxins act at the cholinergic nerve terminals thanks to an efficient and specific mechanism of cell recognition which is based on a dual receptor system that involves gangliosides and protein receptors. Binding to surface-anchored gangliosides is the first essential step in this process. Here, we determined the X-ray crystal structure of the binding domain of BoNT/E, a toxin of clinical interest, in complex with its GD1a oligosaccharide receptor. Beyond confirmation of the conserved ganglioside binding site, we identified key interacting residues that are unique to BoNT/E and a significant rearrangement of loop 1228–1237 upon carbohydrate binding. These observations were also supported by thermodynamic measurements of the binding reaction and assessment of ganglioside selectivity by immobilised-receptor binding assays. These results provide a structural basis to understand the specificity of BoNT/E for complex gangliosides.

## 1. Introduction

The botulinum neurotoxins (BoNT) are among the most toxic molecules known to man and are responsible for botulism, a rare but potentially fatal paralytic disease. They act at the neuromuscular junction by inhibiting the release of presynaptic acetylcholine [1]. These potent toxins are also widely used as treatment for a rising number of medical conditions, including neuromuscular disorders such as cervical dystonia and spasticity, overactive bladder, migraines; as well as for other well-known cosmetic applications [2,3].

There are multiple BoNT serotypes (BoNT/A-G,/X), including natural chimeric variants (e.g., BoNT/DC,/HA) and over 40 subtypes [4,5,6] which have distinct mechanisms of action that result in varying properties in terms of onset, duration of action and potency. More recently, a number of related non-clostridial BoNT-like toxins have also been described (reviewed in [7]), although they do not seem to be toxic towards mammalian species. BoNTs share a common architecture which consists of three functional domains [8]. The binding (H_C_) and translocation domains (H_N_) combined to form the heavy chain (HC) which is linked by a single disulphide bridge to the light chain (LC) following cleavage of the toxin into its active di-chain form. LC is a zinc protease that targets the soluble NSF attachment protein receptors (SNARE) which are responsible for exocytosis [9]. The toxins bind specifically to the cholinergic nerve terminals that promote their uptake in neurons via receptor-mediated endocytosis. The acidic vesicular pH then triggers a conformational change that mediates translocation of LC across the endosomal membrane and into the cytosol, where they can act on their SNARE substrate, thereby inhibiting neurotransmission [10].

The purified botulinum neurotoxins developed commercially for therapeutic and cosmetic applications include serotypes A and B, with variations in their formulation and pharmacological profiles being exploited for different applications [2,3]. In particular, BoNT/A is known for its long-lasting clinical response (3–4 months). More recently, serotype E has been investigated in clinical trials [11,12], since its distinct pharmacological profile may represent an interesting therapeutic alternative for applications where a faster onset and shorter duration of effect is beneficial [13].

BoNT/E recognises neurons via a dual receptor mechanism that involves binding of the H_C_ domain to cell-surface gangliosides [14,15,16] and the synaptic vesicle glycoprotein 2 (SV2) [17,18]. Gangliosides are glycosphingolipids anchored within the plasma membrane that vary in structure depending on their sialic acid (*N*-acetylneuraminic acid) composition, with GM1, GD1a, GD1b, and GT1b representing the majority of mammalian neuronal gangliosides [19]. Ganglioside binding is common to all clostridial neurotoxins and consists of a pocket in the H_C_ domain that holds a conserved SxWY motif [16]. However, each BoNT serotype presents variation in ganglioside specificity, with BoNT/E recognising GD1a and GT1b with higher affinity than GM1 [20,21]. While adherence to the receptor is arranged around the main conserved interaction with the central GalNAc3-Gal4 carbohydrate, surrounding H_C_ residues can also take part in binding and affect specificity [20].

The crystal structure of full-length BoNT/E has been determined previously [22] and showed a unique arrangement where all three domains share a common interface, in a structural organisation that is also associated with a faster translocation rate [23]. In addition, details of the sugar recognition mechanisms are available for several serotypes from the structures of H_C_/A [24],/B[25],/C [26],/D [27] and/F [20] in complex with ganglioside carbohydrate analogues (reviewed in [28]), but not for BoNT/E.

Here, we present the biophysical characterisation of ganglioside binding by BoNT/E, including the crystal structure of Hc/E in complex with the GD1a-oligosaccharide, and a thermodynamic analysis of this interaction by isothermal titration calorimetry (ITC). We also evaluated the carbohydrate selectivity of the toxin by analysing receptor-binding to a range of immobilised gangliosides. These results provide the molecular details of BoNT/E’s specificity for complex gangliosides by identifying significant conformational changes upon receptor-binding, involving residues outside of the conserved ganglioside binding motif. This study may help the design of botulinum neurotoxins with enhanced pharmacological properties.

## 2. Results and Discussion

### 2.1. X-ray Crystal Structure of H_C_/E in Complex with the GD1a-Oligosaccharide

**Figure 1:**
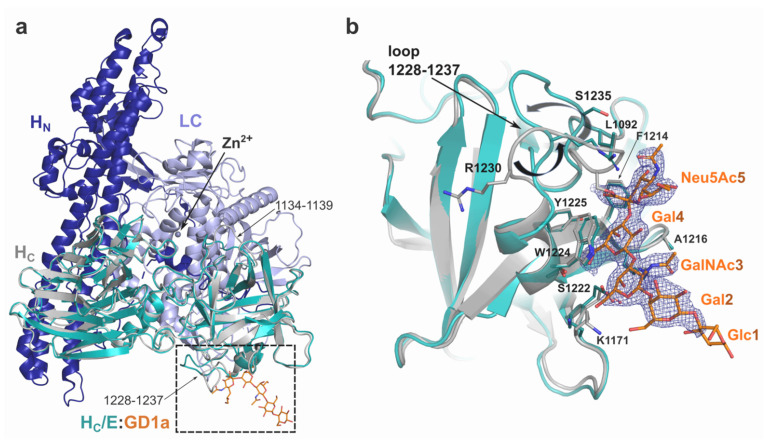
Structure of BoNT/E. (**a**) Superposition of the H_C_/E:GD1a complex structure with full-length BoNT/E (PDB 3FFZ [22]). Domain organisation of BoNT/E is highlighted with the light chain (LC) in light blue, translocation domain (H_N_) in blue and the binding domain (H_C_) in grey. H_C_/E from the complex structure is shown in teal with the GD1a-oligosaccharide in orange. (**b**) Close-up view of the ganglioside binding site. Electron density around GD1a is shown as a blue mesh (2*Fo*-*Fc* map at 1.5 σ). Movement of loop 1228–1237 is represented by black arrows.

The purified H_C_/E protein consisted of residues 820 to 1252 of full-length BoNT/E with a dual N-terminal poly-His and HA tag. Crystals containing H_C_/E in complex with the GD1a carbohydrate were produced in space group P2_1_, which diffracted to a resolution of 2.2 Å (Table 1), with two H_C_/E molecules per asymmetric unit. The GD1a-oligosaccharide fully occupied the receptor-binding pocket of both H_C_/E monomers, however weak electron density around the non-interacting Neu6Ac6 and Glc moieties (Figure 1) means that they could not be included in the model accurately. Additionally, no electron density was observed for the N-terminal region up to residue 848, which includes the protein tags.

#### 2.1.1. Conformational Changes upon Receptor Binding

**Figure 2:**
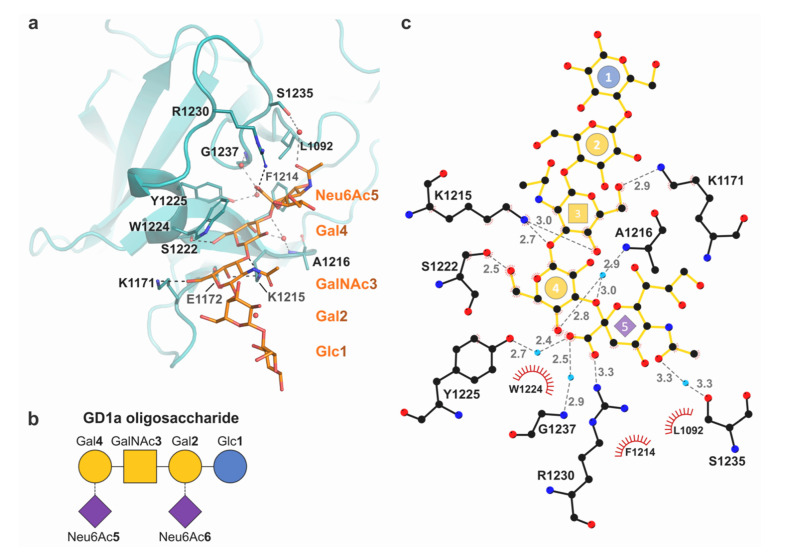
Mechanism of carbohydrate recognition (**a**) Structure of H_C_/E (teal) in complex with the GD1a-oligosaccharide (orange). Molecular interactions are shown with hydrogen bonds and water-mediated contacts as black and grey dashes, respectively, and water molecules as red spheres. (**b**) Glycoblock schematic representation [29] of the GD1a carbohydrate. (**c**) Schematic of ligand interactions (produced with LigPlot+ [30]). Electrostatic bonds are shown in grey dashes with distance in Å. Residues involved in hydrophobic interactions are represented by red arcs.

The overall structure of H_C_/E bound to GD1 is similar to its fold within context of the full-length toxin, with coordinates differing only by a root-mean-square deviation (rmsd) of 0.4 Å (all atoms from 404 aligned residues with PDB ID 3FFZ) (Figure 1). The N-terminal lectin-like subdomain is identical in both structures, while small variations can be observed in two loop regions of the C-terminal β-trefoil subdomain. Of note, loop 1134–1139, which normally interacts with LC as part of a domain arrangement that is unique to BoNT/E [22], shows flexibility on its own. More importantly, a significant conformational change is observed for loop 1228–1237 (Figure 1b, Supplement Video S1) likely triggered by its direct interaction with the sialic acid head group (Neu6Ac5) of GD1a. In particular, R1230 seems to take an important role in this loop movement, as its side chain repositions itself so that the guanidino group makes electrostatic interaction with Neu6Ac5, thus pushing the main chain and rest of the loop further away from the ganglioside. This interaction is also stabilized by water-mediated bridges with residues S1235 and G1237 (Figure 2).

#### 2.1.2. Molecular Details of GD1a Binding

The ganglioside binding site of BoNT/E had previously been described from sequence homology with other BoNTs and mutagenesis studies [14,15,20]. The receptor-bound structure of H_C_/E reveals an overall ganglioside binding mechanism similar to other BoNTs with a central role for the conserved ganglioside binding site (GBS). Three of the five carbohydrate moieties within the GD1a polysaccharide: *N*-acetylneuraminic acid [Neu6Ac5], galactose [Gal4], and *N*-acetylglucosamine [GalNAc3], interact directly with the GBS (Figure 2).

In addition to the electrostatic interactions described above with loop 1228–1237, Neu6Ac5 also forms a water-mediated contact with Y1225 and is further stabilised by hydrophobic interactions with the adjacent L1092 and F1214 residues. Gal4 interacts with the central SxWY motif by a hydrogen bond with S1222, and is held in position by aromatic interactions with Y1225 and stacking against the indole ring of W1224. Gal4 also interacts via a water bridge with the main chain of neighbouring A1216. The side chain of E1272 is within electrostatic distance of both Gal4 and GalNAc3, as the latter also forms strong hydrogen bonds to the side chain of K1215 and K1171. No contacts between H_C_/E and Gal2 or Glc1 were observed. While Neu6Ac6 could not be included in our refined crystallographic model, it cannot be excluded that it might interact weakly with the GBS.

Overall the structure presented here is coherent with previously reported observations that single point mutations E1172A and W1224L abrogate neurotoxicity [15], as these modifications would reduce binding of BoNT/E to the GD1a core sugars (GalNAc3-Gal4). Similarly, mutations at position K1215 to alanine or histidine were observed to hinder binding to GD1a [20], likely by causing the loss of, or disturbing, electrostatic interactions with GalNAc3. Interestingly, while R1230 is seen to contribute significantly to Neu6Ac5 binding and repositioning of loop 1228–1237 in the crystal structure of the H_C_/E:GD1a complex, mutation R1230A was shown to only result in a partial loss of affinity for the receptor [20]. A change to alanine may not affect the larger loop movement which is also driven by water-mediated binding with the downstream S1235 and G1237 residues. Additionally, Benson et al. also explored the potential role of K1093 [20], by analogy with the binding site of H_C_/F in which an arginine at the equivalent position was shown to be in direct contact with Neu6Ac5. However, mutation K1093R did not alter binding, whilst K1093A resulted in a 4-fold increase in affinity for GD1a. It is possible that alanine in that position could extend the hydrophobic pocket formed by the surrounding L1092 and F1214, thus providing additional interaction with the sugar ring of Neu6Ac5.

### 2.2. Thermodynamic Analysis of GD1a Binding

**Figure 3:**
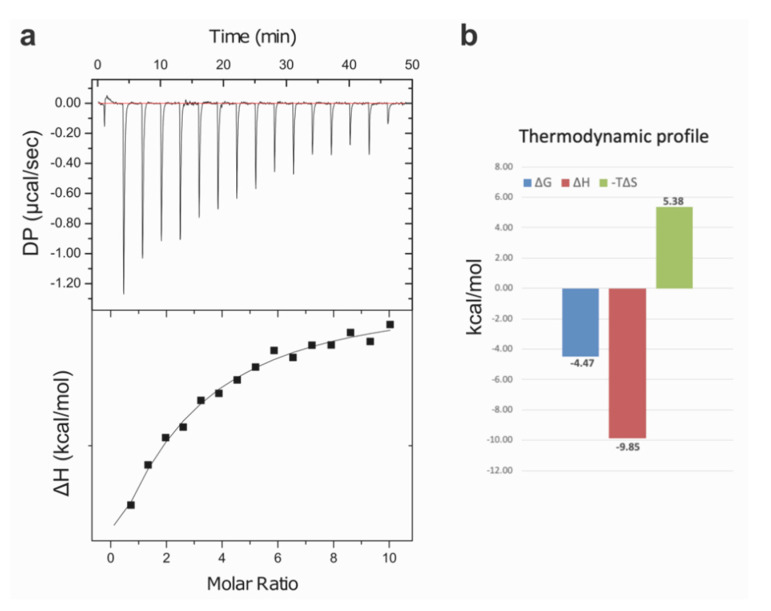
Isothermal Titration Calorimetry analysis (**a**) Example of a titration profile for H_C_/E with the GD1a-oligosaccharide. Top: raw data from the titration at 25 °C (differential power in µcal/s dispersed over time). Bottom: enthalpy of binding (kcal/mol) for each injection plotted against the protein:ligand molar ratio. Data were analysed with a single-site binding model least-squares-fit, resulting with *K_D_* = 530 µM (±40.0) (**b**) Binding free energies (ΔG), enthalpies (ΔH) and entropies (ΔS), in kcal/mol, derived from 3 titrations with the GD1a-oligosaccharide.

ITC experiments were carried out with H_C_/E in order to provide additional information on the molecular details of carbohydrate binding (Figure 3). The binding affinity measured for the GD1a-oligosaccharide, *K*_D_ = 530 µM (±40.0) was consistent with previous report of the weak affinity of H_C_/A (*K*_D_ = 1 mM) [31]. Binding to GM1 or GD1b carbohydrates could not be detected in similar conditions, which confirms observations from immobilised receptor assays [20]. The discrepancy with the stronger affinities reported from a plate-based ganglioside assay, with apparent B_50_ values of 0.23 and 0.6 µM for H_C_/E and/A, respectively, are possibly the results of additional non-specific hydrophobic contributions or interactions with the ganglioside’s ceramide lipophilic tail. Further work is needed to assess the overall binding of full-length BoNTs to membrane-anchored gangliosides, although specificity is most likely driven by recognition of the oligosaccharide parts of the receptor.

Because of the weak interaction affinity, which results in a lack of plateau stages in the titrations, the thermodynamic profile should be interpreted with caution [32]. Nevertheless, H_C_/E binding to the GD1a-oligosaccharide presented a typical enthalpy-driven recognition process. Noticeably, the favourable binding enthalpy (−9.9 kcal/mol) is compensated by significant entropy contribution (−5.4 kcal/mol). These results are consistent with observations from the X-ray crystal structure on the conformational change of loop 1228–1237 upon receptor binding, which also involves a strong network of hydrogen bonds with the Gal4-Neu6Ac5 moieties.

### 2.3. Immobilised Receptor Binding Assays

**Figure 4:**
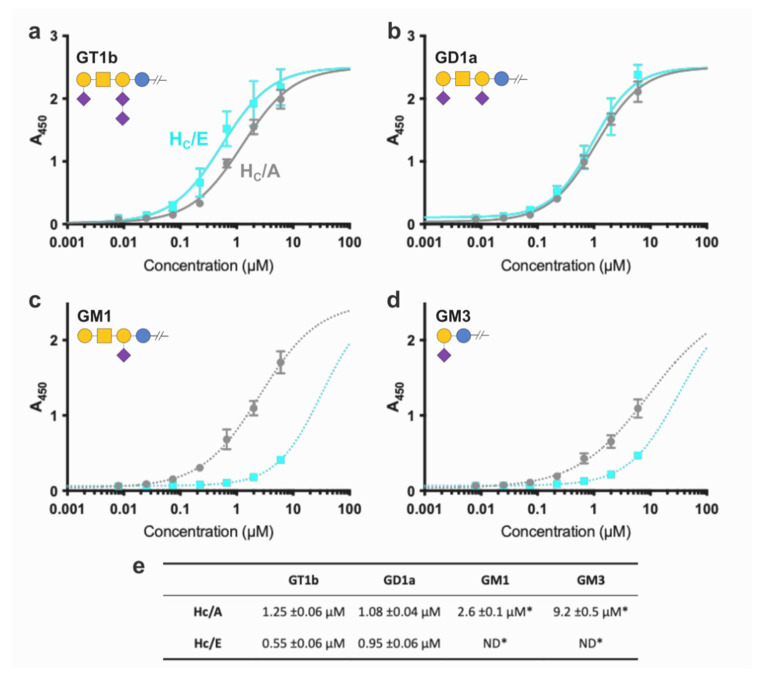
Ganglioside binding. Binding of H_C_/E (cyan), and H_C_/A (grey) to GT1b (**a**), GD1a (**b**), GM1 (**c**), and GM3 (**d**). Assays were performed in triplicate. Glycobloc schematic representation [29] of the ganglioside is also included. (**e**) Apparent K*d* (K*d_app_*) values for each of the fitted binding assays, with standard error. Data marked by (*) were fitted with model parameter limits due to weak binding affinity.

The ganglioside specificity of BoNT/E was analysed and compared to BoNT/A using a previously described format [20] in which FLAG-tagged binding domains were assayed against pure immobilised gangliosides. Overall, the preference of both toxins for more sialylated gangliosides was confirmed to be similar with stronger binding constant for GD1a/GT1b over GM1 and then GM3 (Figure 4). Noticeably, H_C_/A was consistently better than H_C_/E at binding the simpler GM1 and GM3 gangliosides whilst H_C_/E had generally higher affinity for GD1a and GT1b. Binding to GM3, a short precursor molecule with a Gal-Neu6Ac headgroup was only marginal, highlighting the role of the central GalNAc in the recognition of more complex carbohydrates. Additionally, the difference in affinity observed for GD1a between H_C_/E (K*d_app_* = 0.95 µM) and H_C_/A (K*d_app_* = 1.08 µM) was lower than previous report suggested [20] and the binding constants higher (0.23 and 0.6 µM for H_C_/E and/A, respectively [20]). This might be due to differences in reagents and experimental variations. Interestingly H_C_/E showed significantly stronger binding to GT1b (K*d_app_* = 0.55 µM) compared to H_C_/A (K*d_app_* = 1.25 µM), suggesting a potential interaction of the additional Neu6Ac side group with this serotype.

### 2.4. A unique Mechanism of Ganglioside Recognition

**Figure 5:**
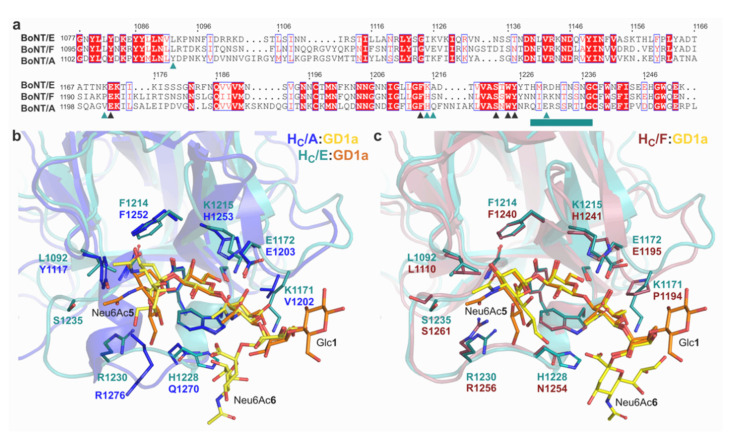
A unique binding site. (**a**) Sequence alignment of the binding domains from BoNT/E,/F and/A, (prepared with ESPript [36]). Identical residues are shown with a red background, and similar residues are boxed. Loop 1227–1238 of BoNT/E is underline (teal). Conserved and unique BoNT/E residues involved in ganglioside binding are marked with black or teal triangles, respectively. (**b**) Superposition of the H_C_/E(teal):GD1a(orange) complex with H_C_/A(blue):GD1a (yellow) from PDB 5TPC [31] and (**c**) with H_C_/F(red):GD1a(yellow) from PDB 3RSJ [20], respectively.

BoNT/E recognises SV2 as its high-affinity membrane protein receptor [17] alongside BoNT/A and/F, however their mechanism of binding for different isoforms can vary depending on the presence of N-glycans on luminal domain 4 of the SV2 binding site, highlighting the importance of carbohydrates for toxin recognition [18,33,34,35]. Previous reports showed that BoNT/E binds to GD1a with a stronger affinity than BoNT/A, but more weakly than BoNT/F [20] which is its closest relative (65% sequence identity). All three toxins share a similar GBS, with the exception of BoNT/E^K1215^ that corresponds to a histidine in types A and F. Noticeably, mutation of BoNT/A^H1253^ and BoNT/E^K1215^ to alanine resulted in a considerable loss of ganglioside binding [20,35] while mutating BoNT/F^H1241^ into a lysine significantly improved binding to both GD1a and GM1a [20]. The weaker affinity of BoNT/E for the GM1a pentasaccharide (Figure 4) suggests that K1215 which interacts with GalNAc3, favors a complementary binding for more complex gangliosides. Indeed, the main differences are located within the area surrounding the GBS (Figure 5). On one side of the receptor binding pocket, K1171 of BoNT/E forms a hydrogen bond with GalNAc3 which further stabilises GD1a within the GBS, whereas this position is occupied by the smaller and hydrophobic valine and proline in BoNT/A and F, respectively. Although the adjacent E1172 is important for ganglioside recognition [15], the loop holding K1171 had not previously been associated with binding in other BoNTs, suggesting that this position contributes significantly to binding of BoNT/E to GD1a, and could be further exploited by including additional electrostatic interactions to increase carbohydrate binding.

On the other side, upon GD1a binding, loop 1228–1237 is seen taking a conformation similar to its equivalent in BoNT/F (residues 1253–264). Remarkably this loop does not need the same receptor-induced movement in that serotype, as it was observed in the same position in both the apoprotein [33] and GD1a-bound structures of H_C_/F [20]. The two serotypes differ considerably in sequence for that loop, which may explain the unique rearrangement seen in BoNT/E that allows for additional electrostatic and solvent-mediated interactions with Neu6Ac5. In BoNT/A, the equivalent position is occupied by a small helix which does not interact with the ganglioside (Figure 5) [31], and may help explain the lower affinity of this serotype compared to BoNT/E and/F [20].

Comparison with the GD1a complexed structure of serotypes A and F also illustrates the position that Neu6Ac6 may take with relation to BoNT/E. Whereas this sialic acid moiety does not appear to be involved in the recognition by BoNT/F, it is within reach of BoNT/A via a hydrogen bond with BoNT/A^W1266^. Although no electron density was observed for this part of the polysaccharide in the H_C_/E:GD1a structure presented here, comparison with BoNT/A suggests it might still be within distance of W1224 or H1228. Interestingly, H1228 is unique to BoNT/E and may help stabilise the GBS via a stacking interaction of its side chain with W1224. BoNT/E^H1228^ might therefore represent an interesting site for investigation to provide additional interactions with Neu6Ac6 and broaden the specificity of BoNT/E for GM1 and GD1b, that are naturally weaker receptors. Furthermore, the higher affinity of BoNT/E for GT1b observed in the immobilised receptor assay also suggests that BoNT/E^H1228^ could interact with an additional Neu6Ac(7) moiety (Figure 4).

Altogether, the data clearly show BoNT/E’s singular recognition strategy which involves movement of loop 1228–1237 upon ganglioside binding in addition to the canonical interaction of the GBS with the core GalNAc3-Gal4 oligosaccharides. It also highlights the important role played by Neu6Ac5, which confirms BoNT/E’s preference for more complex ganglioside such as GD1a and GT1b, over receptors only presenting a Gal4 head group like GM1 and GD1b. Ganglioside binding is usually considered to be the first step in BoNTs neuronal recognition strategy, as the abundance of these glycosphingolipids on the cell surface offer an initial anchor point that promotes binding to the higher affinity membrane protein receptor. Here, we identified the key residues involved in carbohydrate recognition and sites specific to BoNT/E. This study provides the structural basis to design novel BoNT/E molecules with modified affinity or ganglioside selectivity that may alter cell specificity and enhance the pharmacological properties of this serotype, expanding its therapeutic range.

## 3. Materials and Methods

### 3.1. Protein Expression and Purification

Plasmid DNA was kindly provided by Prof. Min Dong (Harvard Medical School, Boston, MA, USA). DNA encoding H_C_/E (strain: D056, BoNT/E1 residues 820 to 1252 [UniProtKB: A8Y875]) was cloned into a modified pET28a(+) vector designed to express proteins containing an N-terminal 6xHis and HA tags with a thrombin protease cleavage site.

H_C_/E was expressed in TB media inoculated using BL21 cells transformed with the vector. Cultures were grown in a LEX bioreactor (Epiphyte3 Inc., Toronto, ON, Canada) at 37 °C. When the OD600 reached 0.8 the temperature was reduced to 18 °C and protein expression induced through the addition of 1 mM IPTG. Cells were grown for a further 18 h before harvesting by centrifugation.

For purification, cells were resuspended in 100 mM HEPES pH 8.0, 500 mM NaCl, 10 mM Imidazole, 10% Glycerol, 0.5 mM TCEP and lysed by pulsed sonication (4s/4s 3 min, 80% amplitude). The lysate was clarified by centrifugation at 50,000× *g* for 30 min before loading onto a pre-equilibrated 5 mL HisTrap HP column (GE Healthcare, Uppsala, Sweden). Purified protein was eluted with 20 mM HEPES pH 7.5, 500 mM NaCl, 500 mM imidazole, 10% glycerol, 0.5 mM TCEP. Fractions containing the target H_C_/E were pooled and further purified using a Superdex200 26/600 column (GE Healthcare, Sweden), pre-equilibrated using 20 mM HEPES pH 7.5, 300 mM NaCl, 10% glycerol, 0.5 mM TCEP. Concentration was performed with Vivaspin filters (10 kDa cut off, Sartorius, Göttingen, Germany). Final concentration was measured by absorbance at 280 nm (NanoDrop Spectrophotometer, ThermoFisher, Uppsala, Sweden) at 13.2 mg/mL, and protein was flash frozen in liquid nitrogen for storage at −80 °C until further use (Supplementary Material: Figure S1).

### 3.2. Isothermal Titration Calorimetry

H_C_/E was first buffer exchanged using a Superdex200 26/600 column (GE Healthcare, Sweden) in 20 mM potassium phosphate pH 7.0, 150 mM NaCl. Binding of the GD1a-oligosaccharide (Elicityl, France; product code GLY098) to H_C_/E was measured via isothermal titration calorimetry on an ITC200 (GE Healthcare, Sweden) at 25 °C and 1000 rpm. A 200-µL solution of H_C_/E at a concentration of 100 µM was added to the cell. Binding was measured upon the addition of GD1a in a stepwise manner, typically 16 injections of 2.5 µL each, at a concentration of 5 mM. The first titration was set to 0.5 µL, and was subsequently deleted in the data analysis. Data analysis was performed using the Origin software provided by the manufacturer. N was set to 1 during fitting, as we know that there is only one binding site. Three titrations were performed, the error reported for the *K*_D_ is the standard deviation. No binding could be measured with GD1b or GM1 (Elicityl, France; product codes GLY096 and GLY099, respectively) using similar settings.

### 3.3. X-ray Crystallography

H_C_/E was recovered from samples used in the ITC experiments with the GD1a-oligosaccharide, and concentrated to 8 mg/mL (Vivaspin filters 10 kDa cut off, Sartorius, Germany). The carbohydrate concentration was adjusted to 5 mM prior to co-crystallisation. Crystals were obtained at 21 °C using the sitting-drop vapour diffusion method where 100 nL of the protein solution was mixed with 100 nL reservoir solution consisting of 0.2 M sodium chloride 0.1 M phosphate/citrate pH 4.2, 20 % *w*/*v* PEG 8000 from the JSCG+ screen (Molecular Dimensions, Sheffield, UK).

X-ray diffraction data were collected from single crystals at 100 K on beamline P13 at the Petra III synchrotron (Germany) using a PILATUS 6M-F detector (Dectris, Baden, Switzerland). Diffraction data were indexed and integrated using XDS [37]. Data were scaled and merged using AIMLESS [38] from the CCP4 suite [39]. An initial model of H_C_/E was generated from PDB 3FFZ [22] for phasing by molecular replacement using Phaser [40]. The working models were refined using REFMAC5 [41] and manually adjusted with COOT. The conformation of the GD1a within the crystallographic model was validated using Privateer [42]. Protein validation was performed with MOLPROBITY [43]. Crystallographic data statistics are summarized in Table 1. The atomic coordinates and structure factors (PDB ID 7OVW) have been deposited in the Protein Data Bank (http://wwpdb.org). Protein structure figures were rendered with PyMOL (Schrödinger, LLC, New York, NY, USA).

### 3.4. Ganglioside-Binding Assay

Gangliosides GM1, GM3, GD1a and GT1b were purchased from Carbosynth (Compton, UK; product codes OG03918, OG16188, OG03917 and OG03923, respectively). H_C_/A and H_C_/E with N-terminal 6xHis and FLAG tags were produced as described previously [44]. Gangliosides were dissolved in DMSO at a stock concentration of 2.5 mg/mL. Stock solutions were diluted in methanol to reach a final concentration of 2.5 μg/mL; 100 μL (0.25 μg) was applied to each well of a 96-well PVC assay plates. After evaporation of the solvent at 21 °C (overnight), the wells were washed (3×) with 200 μL of PBS/0.1% (*w*/*v*) BSA. Nonspecific binding sites were blocked by incubation for 2 h at 21 °C in 200 μL of PBS/2% (*w*/*v*) BSA. Binding assays were performed in 100 μL of PBS/0.1% (*w*/*v*) BSA per well for 2 h at 4 °C containing the H_C_ samples (serial 3-fold dilution ranging from 6 μM to 8 nM). Following incubation, wells were washed 3× with PBS/0.1% (*w*/*v*) BSA and then incubated with monoclonal anti-FLAG HRP-conjugated antibody (Merck, A8592) diluted 1:20,000 into 100 µL PBS containing 0.1% (*w*/*v*) BSA for 1 h at 4 °C. Finally, after three washing steps with PBS/0.1% (*w*/*v*) BSA, bound samples were detected using Ultra TMB substrate solution (ThermoFisher, Waltham, MA, USA, 34,029) (100 μL/well). The reaction was terminated after incubation for 5 min at 21 °C by addition of 100 μL of 1 M sulphuric acid. Absorbance at 450 nm was measured with a Tecan Infinite 200 (Männedorf, Switzerland). Results were analysed with Prism (GraphPad, La Jolla, CA, USA), using a non-linear logistic binding fit.

## Figures and Tables

**Table 1:** Crystallographic Data Collection and Refinement.

	H_C_/E:GD1a-Oligosaccharide
**Data Collection**	
Beamline	PETRA III-P13
Wavelength (Å)	0.976
Space group	P2_1_
Cell dimensions:	
*a, b, c* (Å)	69.4, 84.9, 79.9
α, β, γ (°)	90.0, 91.6, 90.0
Resolution (Å)	53.1—2.2 (2.27—2.20) ^1^
No. total/unique reflections	133072/44975
R_meas_	0.094 (0.748) ^1^
R_pim_	0.066 (0.529) ^1^
CC_1/2_	0.987 (0.807) ^1^
< I/σ(I) >	7.5 (1.7) ^1^
Completeness (%)	95.4 (92.5) ^1^
Redundancy	3.0 (2.8) ^1^
**Refinement**	
R_work_/R_free_	0.22/0.27
*B*-factors (A^2^):	
Protein (all atoms) ^2^	41.5/44.9
GD1a ^2^	57.9/54.1
Solvent	37.5
R.m.s.d. Bond lengths (Å)	0.002
R.m.s.d. Bond angles (°)	1.22
Ramachandran statistics:	
Favoured (%)	96.9
Outliers (%)	0.12
**PDB ID**	7OVW

^1^ Values in parentheses are for highest-resolution shell. ^2^ Values for each molecule of the asymmetric unit.

## Data Availability

The crystallographic data presented in this study are openly available in the Protein Data Bank (https://doi.org/10.2210/pdb7OVW/pdb).

## Supplementary Materials

The following are available online at https://www.mdpi.com/article/10.3390/ijms22158315/s1, Figure S1: SDS PAGE analysis of purified H_C_/E samples. Video S1: Conformational change in H_C_/E upon GD1a receptor binding.
