# Supplementary material for: Mechanism of Ganglioside Receptor Recognition by Botulinum Neurotoxin Serotype E
Source: Int J Mol Sci. 2021 Aug 2;22(15):8315. doi: 10.3390/ijms22158315 (PMC8346984; doi:10.3390/ijms22158315)

Supplementary information

Figure S1. SDS PAGE analysis of H_C/E samples. **(a)** Purified sample used for ITC and crystallisation experiments (lane 1). **(b)** Purified FLAG-tagged sample used for ganglioside-binding assays (lane 2).

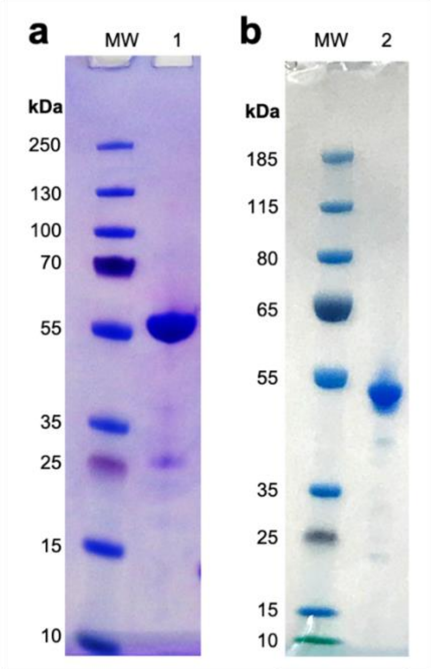

Supplement: Supplementary file 1 [file ijms-22-08315-s001.zip › ijms-1297274-supplementary.pdf]
